# Prevention of Bone Growth Defects, Increased Bone Resorption and Marrow Adiposity with Folinic Acid in Rats Receiving Long-Term Methotrexate

**DOI:** 10.1371/journal.pone.0046915

**Published:** 2012-10-05

**Authors:** Chia-Ming Fan, Bruce K. Foster, Susanta K. Hui, Cory J. Xian

**Affiliations:** 1 Sansom Institute for Health Research, and School of Pharmacy and Medical Sciences, University of South Australia, Adelaide, SA, Australia; 2 Discipline of Paediatrics, University of Adelaide, Adelaide, SA, Australia; 3 Department of Orthopaedic Surgery, Women’s and Children’s Hospital, North Adelaide, SA, Australia; 4 Masonic Cancer Center and Department of Therapeutic Radiology, University of Minnesota, Minneapolis, Minnesota, United States of America; Faculté de médecine de Nantes, France

## Abstract

The underlying pathophysiology for bone growth defects in paediatric cancer patients receiving high dose methotrexate chemotherapy remains unclear and currently there are no standardized preventative treatments for patients and survivors. Using a model in young rats, we investigated damaging effects of long-term treatment with methotrexate on growth plate and metaphyseal bone, and the potential protective effects of antidote folinic acid. This study demonstrated that chronic folinic acid supplementation can prevent methotrexate-induced chondrocyte apoptosis and preserve chondrocyte columnar arrangement and number in the growth plate. In the metaphysis, folinic acid supplementation can preserve primary spongiosa heights and secondary spongiosa trabecular volume by preventing osteoblasts from undergoing apoptosis and suppressing methotrexate-induced marrow adiposity and osteoclast formation. Systemically, plasma of folinic acid supplemented rats, in comparison to plasma from rats treated with MTX alone, contained a significantly lower level of IL-1β and suppressed osteoclast formation *in vitro* in normal bone marrow cells. The importance of IL-1β in supporting plasma-induced osteoclast formation was confirmed as the presence of an anti-IL-1β neutralizing antibody attenuated the ability of the plasma (from MTX-treated rats) in inducing osteoclast formation. Findings from this study suggest that folinic acid supplementation during chronic methotrexate treatment can alleviate growth plate and metaphyseal damages and therefore may be potentially useful in paediatric patients who are at risk of skeletal growth suppression due to chronic methotrexate chemotherapy.

## Introduction

Bone growth involves the production of calcified cartilage in the growth plate, which will be first converted into woven spongy bone (composed of bony trabeculae of mineralized cartilage cores in the primary spongiosa region) and then further modeled and remodeled into lamellar trabecular bone (secondary spongiosa) in the metaphysis [1]. These processes rely on the regulation of growth plate chondrocyte proliferation, differentiation and apoptosis, and differentiation and function of bone-forming cells (osteoblasts) and bone resorptive cells (osteoclasts). Any disruption to these carefully controlled cell processes (collectively termed endochondral ossification) will result in bone growth defects. With the development of more successful chemotherapy and improved childhood cancer survivor rates, chemotherapy-associated bone growth abnormalities in survivors of paediatric cancers, including bone growth arrest, fractures, osteopenia and osteoporosis, have become significant clinical problems [2], [3], [4], [5]. Typically, bone metabolism in children with acute lymphoblastic leukemia (ALL) (the predominant childhood cancer) is known to be disturbed after chemotherapy, resulting in reduced bone lengthening and bone loss. Since bone growth defects or bone loss during childhood may predispose to osteopenia and osteoporosis in later life, it is important to understand the mechanisms of chemotherapy-induced bone damage to the growing bones and develop strategies for prevention of such damages.

Methotrexate (MTX) is prescribed widely for treatment of both cancers (particularly for ALL and osteosarcoma) and rheumatoid arthritis (RA). MTX acts by inhibiting the enzyme dihydrofolate reductase, thus blocking thymidylate and purine synthesis [6]. While many studies have examined effects of long-term low-dose MTX on bone metabolism and have reported no significant adverse effects on bone mineral density (BMD) [7], [8], [9], long-term intensive chemotherapy with MTX has been shown to cause serious damage to bone development in paediatric patients [10], [11]. Despite some previous studies showing that MTX chemotherapy can suppress skeletal growth by disrupting growth plate structure and functioning of bone cells [12], [13], [14], how long-term high-dose MTX chemotherapy (used typically in ALL treatment) causes bone growth defects remains unclear. In addition, due to high success rates and the intensified use of chemotherapy in children (particularly with ALL that account for almost one third of childhood cancers and having a survival rate of over 80%), it is important to develop potential strategies for protecting bone growth during MTX chemotherapy. Folinic acid (FA), an antidote that has been clinically used to reduce toxicity of high dose MTX in soft tissues, has been recently shown to have protective effects in the skeleton of young rats receiving acute MTX chemotherapy [13]. However, no long-term studies have examined potential protective effects of FA in chemotherapy-induced bone damages. Using a chronic model in young rats mimicking the MTX regimen in treating ALL, the current study examined the structural, cellular and molecular damages to the bone growth unit (growth plate and metaphysis) caused by long-term high-dose MTX treatment, as well as the potential protective effects of FA supplementary treatment.

## Materials and Methods

### Animal Trials and Specimen Collection

**Figure 1:**
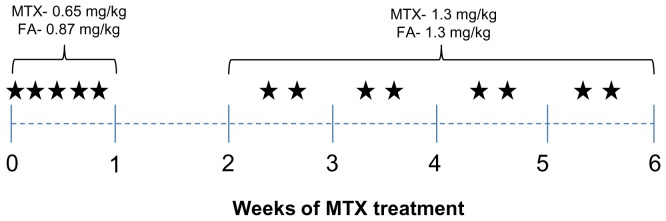
Dosing schedules for 6 weeks with the induction and maintenance phases of MTX chemotherapy. *indicates one injection of MTX with or without folinic acid (FA).

Groups of young male Sprague-Dawley rats of 5 weeks old were randomly allocated into three treatment groups receiving saline, MTX, or MTX plus FA (MTX+FA) (n = 6/group). MTX was administered in two phases (**Fig. 1**) mimicking regimen for ALL treatment [15]. During the induction phase, rats were subcutaneously injected with MTX daily at 0.65 mg/kg for 5 consecutive days, followed by 9 days of rest. FA was injected intraperitoneally 6 hours after MTX at 0.87 mg/kg. During the maintenance phase (from day 15 or week 2 to week 6), rats received MTX at 1.3 mg/kg twice weekly with or without FA at 1.3 mg/kg. By the end of week 6, rats were sacrificed humanely using carbon dioxide overdose (**Fig. 1**). Ninety minutes prior to sacrifice, rats were injected with 5′-bromo-2′-deoxyuridine (BrdU) (Sigma, NSW, Australia) at 50 mg/kg to label S-phase nuclei for studying cell proliferation. The above protocols were approved by Women’s and Children’s Hospital Animal Ethics Committee (South Australia).

Blood samples were collected for plasma used in analysis for its ability in inducing osteoclast formation *in vitro* or in cytokine ELISA assays. Left tibias were fixed in 10% formalin, decalcified in Immuocal solution (Decal Corporation, Tallman, NY), processed and embedded in paraffin for 4 µm thick sections for histological analyses. Left femurs were stored at −80°C until used for micro-computed tomography (µ-CT) analysis. From the right tibia, growth plate cartilage and metaphyseal bones were collected for gene expression analysis.

### Histological Analysis of Growth Plate and Metaphyseal Bone Structure

Paraffin sections were dewaxed and stained with 0.3% Alcian blue in 3% acetic acid followed by haematoxylin and eosin (H&E) staining. In the growth plate, stained sections were used for morphometric measurements of growth plate zonal heights and columnar chondrocyte counts (cells/mm length). In the metaphysis region, primary spongiosa heights, osteoblast and adipocyte density measurements were made as described [8], [14], [16]. Briefly, primary spongiosa heights were obtained by measuring the heights between the end of growth plate and the top of secondary spongiosa. Osteoblast density was obtained by counting cuboidal mononuclear cells along the trabecular surface in 6 sequential images along primary spongiosa as described [8], [14], and expressed as osteoblasts number per mm^2^ trabecular area. Adipocytes within bone marrow area were counted in 4 random images within the lower secondary spongiosa region, and expressed as adipocyte number per mm^2^ marrow area. These measurements were made on 3 separate sections of 200 µm interval.

### Ex-vivo Micro-computed Tomography (µ-CT) and Analysis of Bone Parameters

Left femurs were used for examining treatment effects on overall trabecular bone volume and trabecular bone structures by a µ-CT scanner (Skyscan 1076: Skyscan, Antwerp, Belgium), with the scanner equipped at a 20–100 kV X-ray source and a spot size of <5 µm. Using the Skyscan software, a 1.0 mm aluminium filter was selected for scanning rat bones. Other settings were made in the scan dialog box with details of scan width of 35 mm, resolution of 9 µm pixel, rotation step size 0.8 and averaging of 4 for noise reduction in order to achieve better imaging. The following parameters were measured at a high energy scan in the metaphysis: trabecular bone volume/total volume ratio (BV/TV %), trabecular thickness (mm), number (per mm) and separation (mm).

### BrdU Labeling and in situ TUNEL Labeling of Growth Plate Chondrocytes

To examine the treatment effects on growth plate chondrocyte proliferation, BrdU labeling was performed as described [8], [16]. BrdU^+^ cells were counted within the proliferative zone (cells/mm^2^). To examine effects on chondrocyte apoptosis, terminal deoxynucleotidyl transferase dUTP nick end labeling (TUNEL) (Roche Applied Science, NSW, Australia) was performed on sections as instructed. Apoptotic cell density was expressed as apoptotic cells/mm^2^ growth plate area (excluding cartilage/bone transitional zone where the physiological apoptosis of hypertrophic chondrocytes usually occurs).

### In situ DNA Nick Translation for Labeling Apoptosis of Osteoblasts

To examine treatment effects on the apoptosis of osteoblastic cells, *in situ* nick translation (ISNT) reaction was conducted to label the presence of fragmented DNA by direct incorporation of labeled nucleotide in free 3'-OH termini using a nick translation mixture (Roche, NSW, Australia). DNA strand breaks were then detected by anti-Dig-peroxidase (Roche) and DAB reaction (DAKO, CA). Osteoblast apoptosis was quantified within both primary and secondary spongiosa along the surface of trabecular bone, and expressed as apoptotic osteoblasts/mm^2^ trabecular area.

### Quantitative RT-PCR Analysis of Gene Expression

Real time RT-PCR was used to examine treatment effects on mRNA expression of some major matrix proteins and regulatory genes, including growth plate cartilage protein collagen-II, molecules involved in controlling apoptosis (Bcl-2, Bax, Fas, and Fas-L), osteoblast differentiation (Osterix, Osteocalcin), osteoclast differentiation (RANKL, OPG, TNF-α) and adipogenesis (PPARγ). Briefly, total RNA from the growth plate and metaphysis was extracted with TRI reagent (Sigma) then DNase treated with TURBO DNA-*free*™ Kit (Ambion, Austin, TX) for the removal of any contaminating DNA. Samples with an A260/A280 ratio of 1.8 or above were used to synthesize single stranded cDNA using High Capacity RNA-to-cDNA Kit (Applied Biosystems, Sydney, Australia). SYBR Green PCR assays (Applied Biosystems) for each target molecule and reference gene Cyclophilin A (Cyc-A) were performed using primers **(** **Table 1** **)** [16], [17], [18] in triplicate on cDNA samples. PCR assays were run on a 7500 Fast Real-Time PCR System (Applied Biosystems). Analysis of gene expression was done using the comparative Ct (2^−ΔCT^ ) method [17].

**Table 1:** Primer sequences used in this study.

Gene	Forward Primer	Reverse Primer
Cyclophilin-A	GAGCTGTTTGCAGACAAAGTTC	CCCTGGCACATGAATCCTGG
Collagen-IIa	GGGCTCCCAGAACATCAGCTACCA	TCGGCCCTCATCTCCACATGATTG
Bcl-2	CAGCATGCGACCTCTGTTTG	TCTGCTGACCTCACTTGTGG
Bax	CGAGAGGTCTTCTTCCGTGT	GAGCACCAGTTTGCTAGCAA
Fas	AAGATCGATGAGATCGAGCACA	AAGCTTGACACGCACCAGTCT
Fas-L	GAGCTGTGGCTACCGGTGATAT	ACTCACGGAGTTCTGCCAGTTC
RANKL	CCGTGCAAAGGGAATTACAAC	GAGCCACGAACCTTCCATCA
OPG	CACAGCTCGCAAGAGCAAACT	ATATGCCGTTGCACACTGCTT
TNF-α	ATGGCCCAGACCCTCACACTCAGA	CTCCGCTTGGTGGTTTGCTACGAC
PPARγ	AACGTGAAGCCCATCGAGGACATC	CTTGGCGAACAGCTGGGAGGAC

### Measurement of RANKL and Pro-inflammatory Cytokines in Plasma

Enzyme-linked immunosorbent assay (ELISA) was performed to measure osteoclastogenic cytokines in plasma of treated vs. control rats using ELISA kits for TNF-α (BD Biosciences, San Diego, CA), RANKL (R&D Systems, Minneapolis, MN) and IL-1β (R&D Systems).

### In vitro Osteoclast Formation Induced by Plasma from Treated Rats

To determine whether circulating factors may play a role in osteoclastogenesis after long-term MTX treatments, *in vitro* osteoclast formation assay was performed to examine whether plasma derived from treated rats could induce osteoclast formation from bone marrow cells of normal rats. Briefly, non-adherent hematopoietic cells isolated from normal rats were plated in 96-well trays at the density of 3×10^5^ cells/well in triplicate, and cultured overnight in α-MEM media containing 50 µg/ml Pen/Strep, 15 mM HEPES, 10 ng/ml macrophage-colony stimulating factor (M-CSF) (Peprotech, Rocky Hill, NJ), and 10% plasma from control rats or treated rats. Cells were then fed the next day with similar plasma-containing media excluding M-CSF and any other exogenous osteoclastogenic factors except RANKL for the positive control (M-CSF+RANKL). To determine whether IL-1β plays a role in plasma-induced osteoclast formation, a neutralizing antibody against IL-1β (R&D Systems) at two different concentrations (0.04 and 0.08 ng/ml) was added to the plasma-containing media as described [19]. A normal goat IgG control IgG was also used at the same concentrations as the neutralizing IL-1β antibody. In addition, a positive control was included alongside for comparison with the osteoclast formation induced by 10 ng/ml M-CSF and 30 ng/ml RANKL (Peprotech) in the absence of any additional rat plasma as described [8]. Cell culture ended at day 5, and cells were fixed in 4% formaldehyde and TRAP-stained for osteoclasts [8]. To aid identifying osteoclasts, cells were counterstained with Hoechst 33258 fluorescent dye (Invitrogen) for visualization of nuclei. TRAP^+^ cells containing three or more nuclei were identified as osteoclasts. Results were expressed as percentage of osteoclast formation induced by the positive control (M-CSF+RANKL treated culture) as described [19].

### Statistics

Data are presented as means ± SEM and analysed by one-way analysis of variance (ANOVA) with GraphPad Prism 6 Software. When the significance levels (P<0.05) were achieved, a post hoc analysis of groups was performed using a Tukey’s test. Significant values are indicated as follows: * = P<0.05, ** = P<0.01, *** = P<0.001. All measurements were done with n = 6 rats per treatment group.

## Results

### Treatment Effects on Growth Plate Structural Changes and Chondrocyte Proliferation

**Figure 2:**
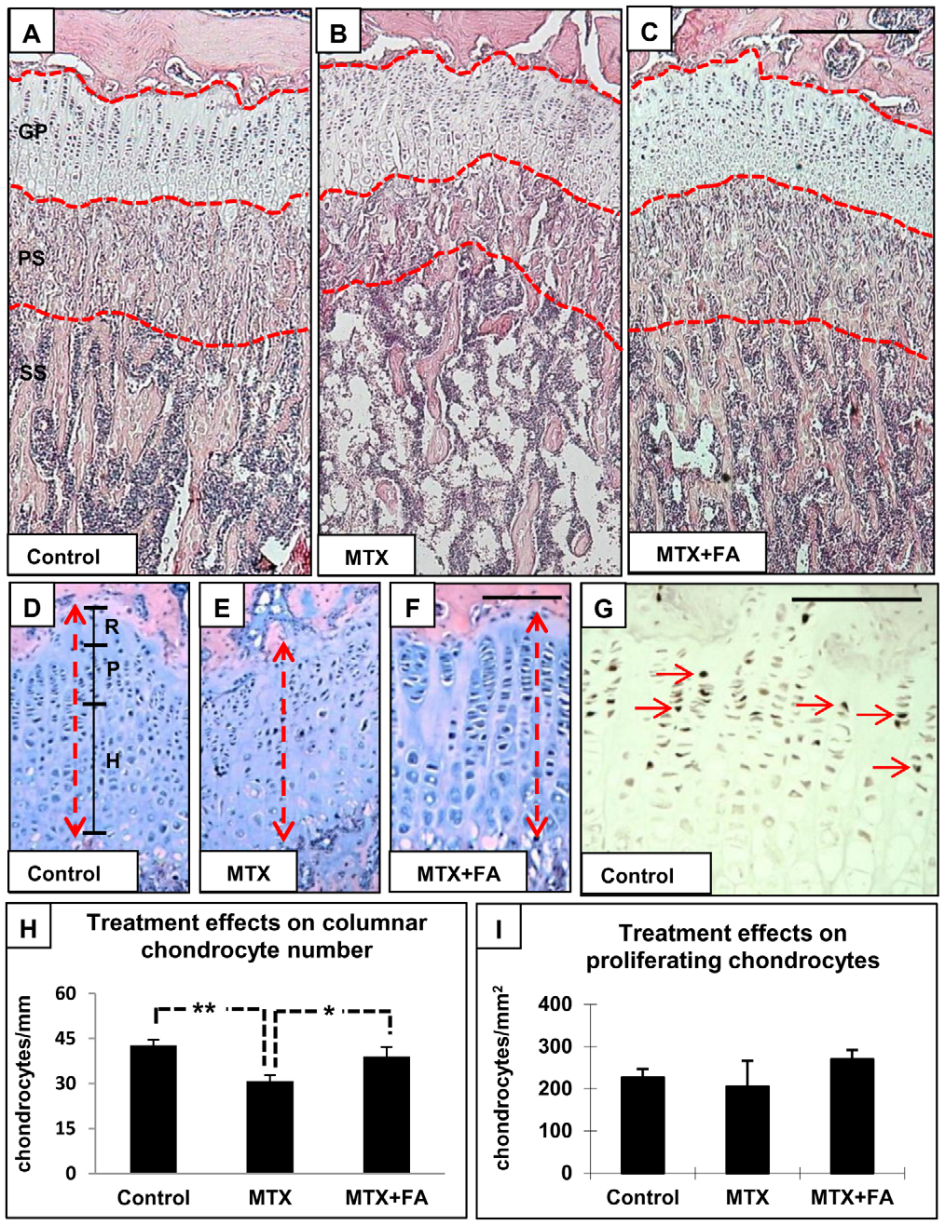
Effects of treatments with MTX alone or with supplementary folinic acid (FA) for 6 weeks on overall structures of rat tibia. H&E staining of rat tibia from control (A), MTX-treated (B) and MTX+FA treated rats (C) at week 6. GP = growth plate, PS = primary spongiosa, SS = secondary spongiosa. H&E staining of growth plates from week 6 control (D), MTX-treated (E) and MTX+FA (F) treated rats. R = resting zone, P = proliferative zone and H = hypertrophic zone. (G) BrdU labeling (arrows) of chondrocytes at proliferative zone of growth plate. (H) Treatment effects on columnar chondrocyte number, which were measured along the red dotted line. (I) Treatment effects on growth plate chondrocyte proliferation. Scale bars on panels A–C = 500µm, D–F = 250 µm and G = 150 µm.

Overall, chronic treatment with MTX alone or together with supplementary FA caused no obvious structural changes to the growth plate **(** **Fig. 2A–2C** **)**. Histomorphometric measurements also revealed no differences in both zonal and total growth plate thicknesses between all treatment groups **(** **Fig. 2D–2F** **)**. However, counting of cells per chondrocyte column revealed a significant reduction of chondrocyte numbers after long-term MTX treatment compared to control rats (P<0.01), while supplementary treatment with FA partially preserved the chondrocyte numbers (P<0.05 vs. MTX) **(** **Fig. 2H** **)**. Apart from the significant reduction in chondrocyte numbers observed, chondrocytes in MTX-treated rats appeared to have disrupted columnar arrangement when compared to control or FA-supplemented rats. However, BrdU labeling for chondrocyte proliferation revealed no differences in density of BrdU^+^ cells between all treatment groups **(** **Fig. 2G and 2I** **)**. In addition, RT-PCR analysis revealed no significant changes in mRNA expression of the major cartilage protein collagen-II (data not shown).

### Treatment Effects on Chondrocyte Apoptosis and Expression of Apoptosis-regulatory Genes

**Figure 3:**
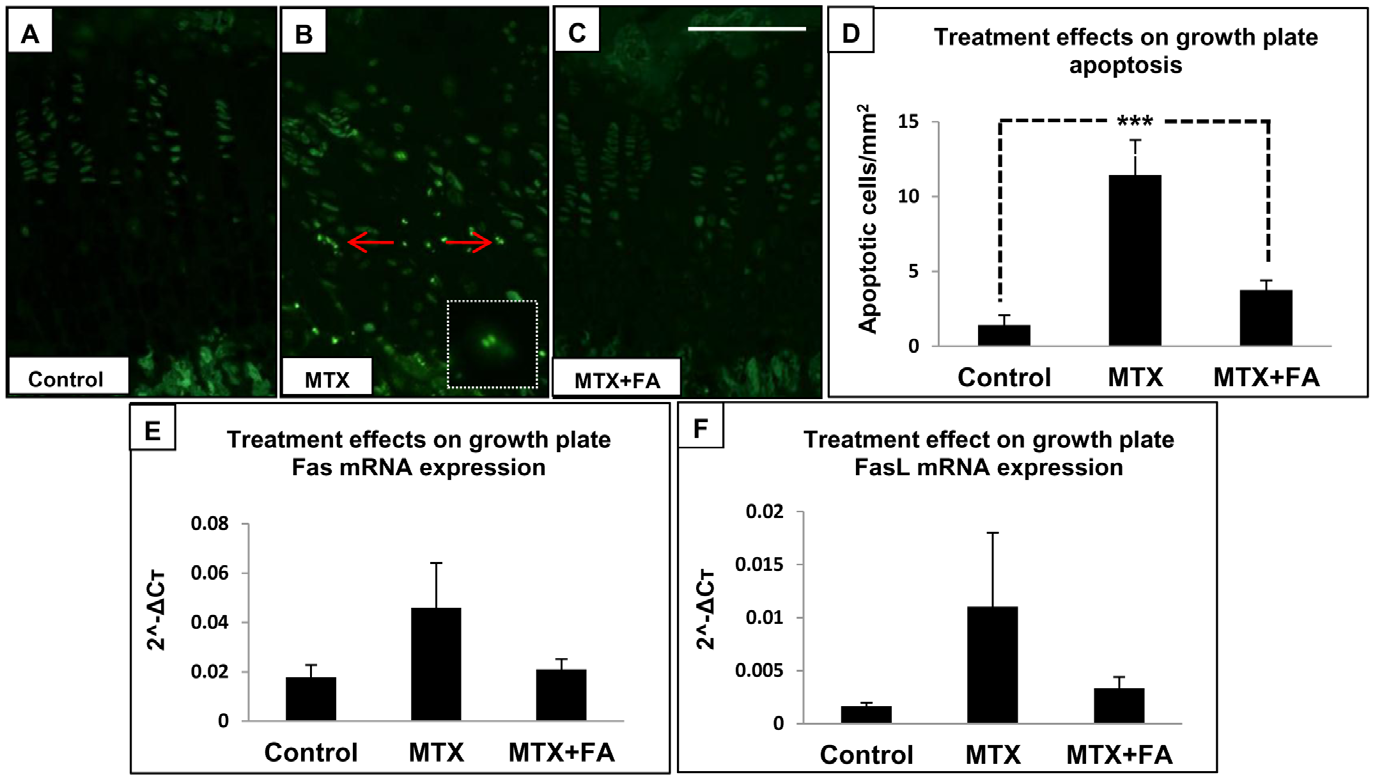
Effects of treatments with MTX alone or with supplementary folinic acid (FA) for 6 weeks on growth plate chondrocyte apoptosis. *In Situ* TUNEL labeling of growth plate apoptotic chondrocytes from week 6 control (A), MTX-treated (B), and MTX+FA (C) treated rats, with arrows pointing to TUNEL positive apoptotic chondrocytes, characterized by fragmented nuclei (enlarged version shown in dotted square). (D) Treatment effects on density of apoptotic chondrocytes in growth plate. (E, F) Treatment effects on mRNA expression of apoptotic death receptor pathway genes Fas and Fas-L relative to Cyclophilin-A. Scale bar on panels A–C = 200 µm.

Apoptotic cells chondrocytes are normally found at the growth plate-bone transitional region, but rarely identified within the growth plate proliferative and hypertrophic zones **(** **Fig. 3A** **)**. After 6 weeks of high-dose MTX treatment, a significant induction of chondrocyte apoptosis was observed within the growth plate in both the proliferative and hypertrophic zones as shown by fluorescent TUNEL positive staining and density quantification (cells/mm^2^) (P<0.001 vs. controls) **(** **Fig. 3B & 3D** **)**. On the other hand, FA supplementation significantly suppressed MTX-induced chondrocyte apoptosis (P<0.001) **(** **Fig. 3C & 3D** **)**.

Quantitative RT-PCR analysis was used to examine expression of apoptosis regulatory genes involved in both mitochondrial pathway (Bcl-2 and Bax) and death receptor pathway (Fas and Fas-L) [20], [21]. While levels of expression of both anti-apoptosis gene Bcl-2 and pro-apoptosis gene Bax were not affected by treatments (data not shown), expression of both death receptor Fas and its ligand (Fas-L) showed a trend of up-regulation in MTX-treated rats, which was not seen in rats receiving supplementary FA treatment **(** **Fig. 3E & 3F** **)**. These results suggest that the death receptor pathway may possibly contribute towards MTX-induced chondrocyte apoptosis, which may be prevented by FA supplementation.

### Treatment Effects on Structural Changes in Metaphysis

**Figure 4:**
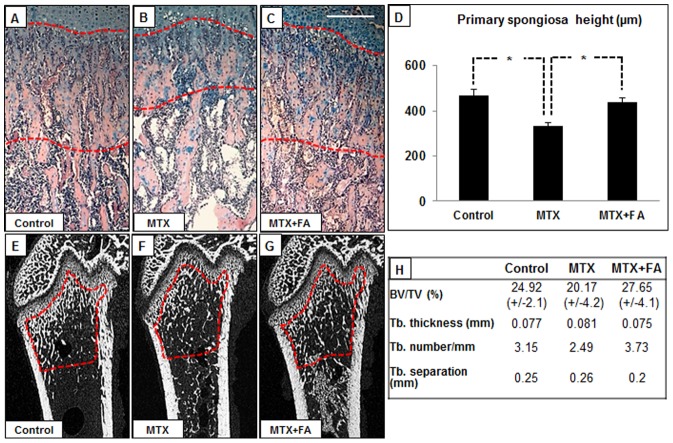
Effects of treatments with MTX alone or with supplementary folinic acid (FA) for 6 weeks on metaphysis structure. H&E alcian staining of metaphyseal bone in control (A), MTX-treated (B) and MTX+FA (C) treated rats, with dotted lines marking the primary spongiosa zone. (D) Treatment effects on primary spongiosa height. μ-CT longitudinal cross-sections of femur from a control (E), MTX-treated (F), and MTX+FA (G) treated rat, with area traced in red dotted lines being used for measurement of trabecular bone volume and structures. (H) Treatment effects on trabecular bone volume and structural changes. BV/TV = Bone volume/Tissue volume, Tb = Trabecular. Scale bar on panels A–C = 250 µm.

Metaphysis is made up with primary and secondary spongiosa, where the primary spongiosa consists of the mineralized cartilage derived from the growth plate, while secondary spongiosa is composed of enlarged mineralized bony trabeculae modeled or remodeled from the primary spongiosa. Histomorphometric analysis of the metaphyseal bone revealed a significant reduction in primary spongiosa heights after 6 week of high-dose MTX treatment (P<0.05 vs. controls), while FA supplementation was able to prevent this reduction **(** **Fig. 4A–4D** **)** (P<0.05 vs. MTX). Further examination of structural changes in metaphysis with µ-CT revealed that the MTX treatment caused an insignificant reduction in trabecular bone volume (BV/TV%) when compared to control rats (20.17±4.2 vs. 24.92±2.1%), and that FA supplementation was able to prevent this bone loss (27.65±4.1 for MTX+FA vs. 20.17±4.2% for MTX alone) **(** **Fig. 4E–4H** **)**. In addition, when compared to control rats, long-term high-dose MTX treatment caused a non-significant reduction in trabecular number (2.49 vs. control 3.15/mm), a slight increase of trabecular thickness (0.081 vs. 0.071 mm) and trabecular separation (0.26 vs. 0.25 mm) **(** **Fig. 4H** **)**. When compared to MTX alone group, FA supplementation resulted in a greater number of trabeculae (3.73 vs. 2.49/mm) and a reduction of trabecular spacing (0.20 vs. 0.26 mm) **(** **Fig. 4H** **)**. Consistent with these µ-CT findings, quantitative histomorphometric analysis on H&E stained bone sections obtained similar results on trabecular bone volume and trabecular structure, with more obvious changes observed in secondary spongiosa (data not shown).

### Treatment Effects on Osteoblast Density and Osteoblast Apoptosis

**Figure 5:**
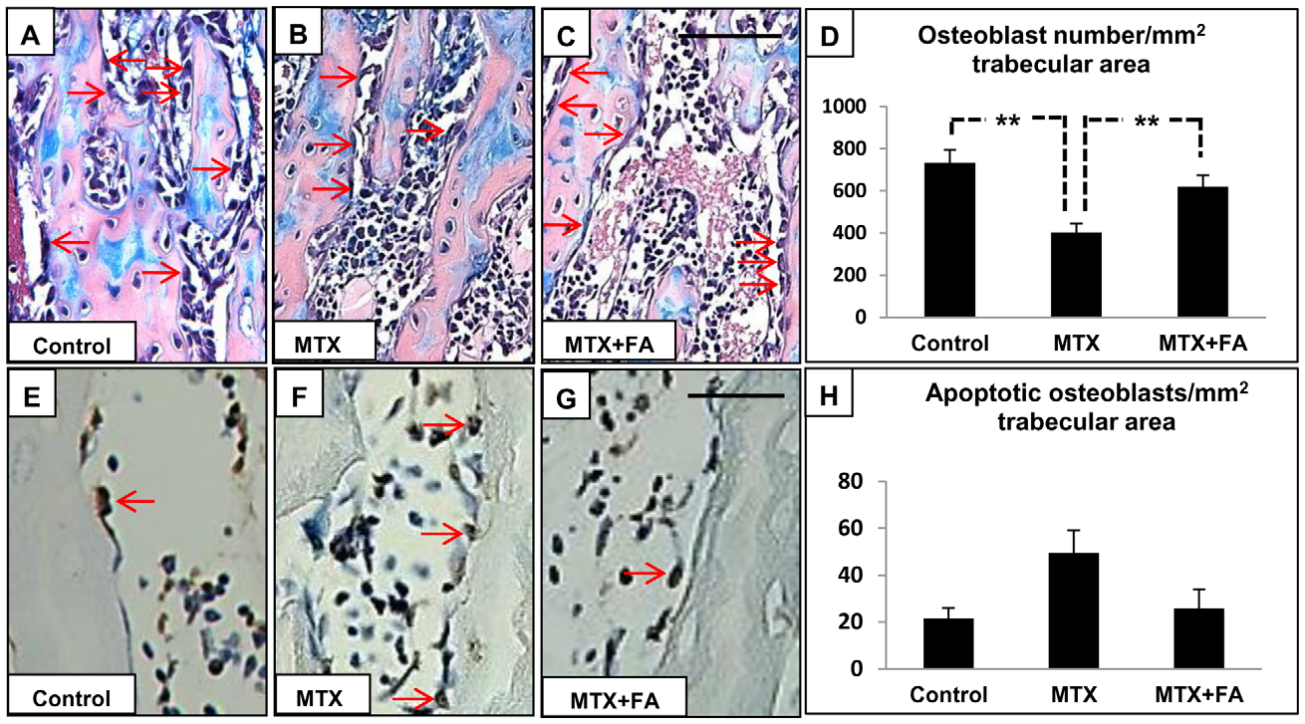
Effects of treatments with MTX alone or with supplementary folinic acid (FA) for 6 weeks on osteoblast density and apoptosis. H&E-stained sections of a tibia from a control (A), MTX-treated (B) and a MTX+FA (C) treated rat, with arrows pointing to osteoblasts. (D) Treatment effects on osteoblast density in primary spongiosa. *In Situ* Nick Translation labeling detecting apoptotic osteoblasts (pointed by arrows) in metaphysis bone from a control (E), MTX-treated (F), and a MTX+FA (G) treated rat. (H) Treatment effects on osteoblast apoptosis in metaphysis bone. Scale bars on panels A–C = 100 µm and E–G = 125 µm.

Trabecular bone volume and structure are directly influenced by numbers and activity of osteoblasts. Counting of osteoblasts on H&E stained sections revealed that high-dose MTX treatment significantly reduced osteoblast density in the primary spongiosa but not in the secondary spongiosa when compared to control rats (P<0.01), while FA supplementation significantly preserved the osteoblast density (P<0.01 vs. MTX) **(** **Fig. 5A–5D** **)**. To determine mechanisms for treatment-induced changes in osteoblast density, treatment effects on osteoblast proliferation and apoptosis were assessed. While BrdU labeling revealed no obvious changes in osteoblast proliferation (data not shown), apoptosis analysis by *in situ* Nick Translation labeling revealed that long-term high-dose MTX treatment caused a trend of more apoptosis among osteoblasts in metaphysis **(** **Fig. 5E, 5F & 5H** **)**. Interestingly, FA supplementary treatment appeared to prevent the MTX-induced osteoblast apoptosis in both primary and secondary spongiosa **(** **Fig. 5G & 5H** **)**.

### Treatment Effects on Osteoclast Density and Expression of Genes Regulating Osteoclastogenesis

**Figure 6:**
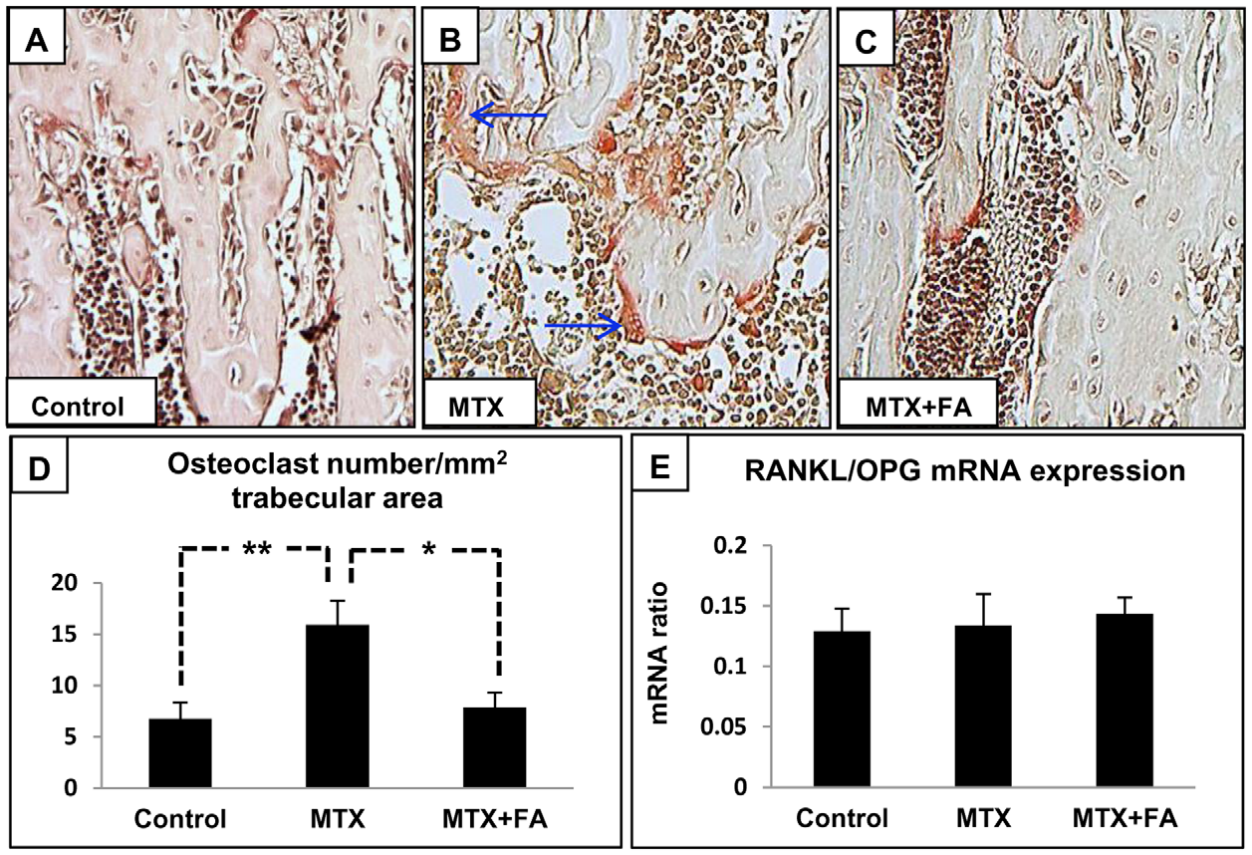
Effects of treatments with MTX alone or with supplementary folinic acid (FA) for 6 weeks on osteoclasts. TRAP-stained sections of tibia from a control (A), MTX-treated (B), and a MTX+FA treated rat (C), with arrows pointing to multinucleated TRAP^+^ osteoclasts. (D) Treatment effects on osteoclast density in primary spongiosa. (E) Treatment effects on metaphyseal bone RANKL/OPG mRNA expression ratio as determined by real time RT-PCR analysis. Scale bar on panels A–C = 200 µm.

Counting of TRAP^+^ osteoclasts adhering on trabecular surface revealed that MTX treatment significantly increased osteoclast density in the primary spongiosa (P<0.01 vs. controls), while supplementary FA treatment significantly suppressed this increase (P<0.05 vs. MTX) **(** **Fig. 6A –6D** **)**. As a mean to examine expression of key genes promoting osteoclastogenesis locally in bone, RT-PCR analysis revealed no significant changes in RANKL/OPG mRNA expression ratio **(** **Fig. 6E** **)** and levels of pro-inflammatory cytokines including TNF-α, IL-1β and IL-6 in the metaphysis (data not shown).

**Figure 7:**
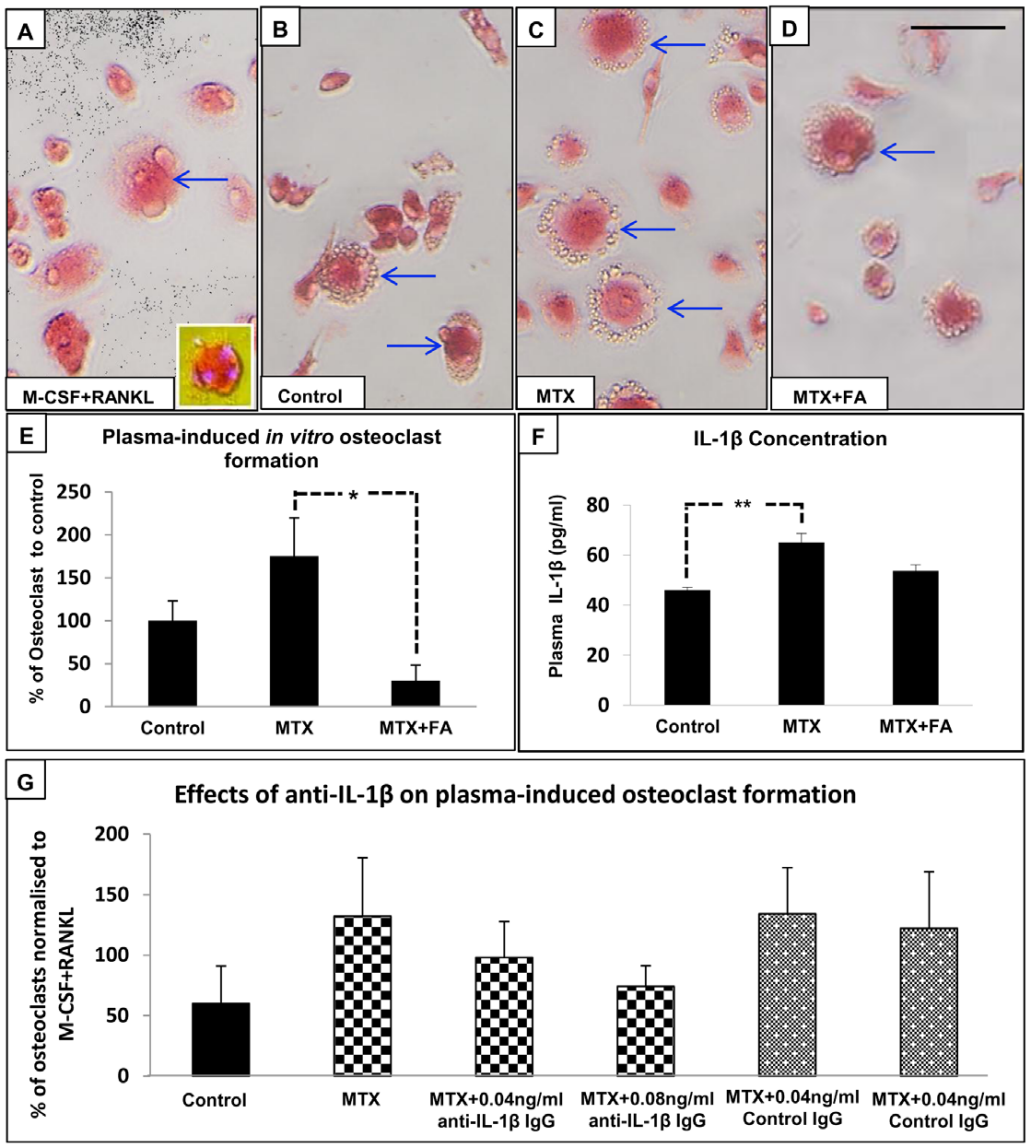
Osteoclast formation in vitro as induced by plasma from rats treated with MTX alone or with supplementary folinic acid (FA) for 6 weeks. *In vitro* osteoclast formation from normal rat bone marrow cells as induced by M-SCF+RANKL (A), or by plasma alone obtained from rats treated for 6 weeks with saline control (B), MTX (C), or MTX+FA combination (D), with arrows pointing to multinucleated TRAP^+^ osteoclasts formed. Hoechst stain was performed to aid visualising nuclei of TRAP^+^ cells (in square). (E) Comparison of osteoclast formation as induced by plasma from rats of different treatment groups, with results expressed as percentage of osteoclasts induced by M-CSF+RANKL (positive control). (F) Plasma concentrations of IL-1β at week 6. (G) Effects of the presence of a IL-1β neutralizing antibody or control IgG at different concentrations on plasma-induced osteoclast formation with plasma from control or MTX-treated rats, with results expressed as percentage of osteoclasts induced by M-CSF+RANKL. Scale bar on panels A–D = 100 µm.

Potential systemic effects of treatment were investigated by examining ability of the plasma alone from treated and control rats (without exogenous M-CSF and RANKL added) in promoting osteoclast formation from normal bone marrow cells. *In vitro* osteoclast formation assays revealed that plasma obtained from MTX-treated rats was able to induce more osteoclast formation when compared to plasma from control rats and positive control with exogenous M-CSF and RANKL, despite statistically insignificant. However, plasma obtained from MTX+FA treated rats induced significantly fewer osteoclasts compared to plasma from MTX-treated rats (P<0.05) **(** **Fig. 7A–7E** **)**. To identify potential circulating osteoclastogenic factors, ELISA assays revealed no obvious differences in TNF-α and RANKL concentrations in plasma obtained from both treated and control rats (data not shown). However, IL-1β concentration in the plasma was significantly increased following 6-week MTX administration (P<0.01 vs. controls) **(** **Fig. 7F** **)**, and FA supplementary treatment was found to partially suppress MTX-induced increase in IL-1β level **(** **Fig. 7F** **)**. To investigate a role of IL-1β in plasma-induced osteoclast formation *in vitro*, the osteoclastogenesis assay was also performed in the presence of a neutralizing antibody against IL-1β as well as a control antibody. Despite statistically being insignificant, the ability of enhancing osteoclast formation by plasma from MTX-treated rats was found to be reduced in the presence of the anti-IL1-β neutralizing antibody in a dose-dependent manner, while the control IgG at both doses did not reduce osteoclast formation induced by plasma from MTX-treated rats, demonstrating specificity of the effects of the anti-IL1-β neutralizing antibody observed **(** **Fig. 7G** **).** Taken together, these data suggest that MTX chemotherapy-induced increase in plasma IL-1β may play a role in inducing osteoclast formation systemically.

### Treatment Effects on Bone Marrow Adipocyte Density

**Figure 8:**
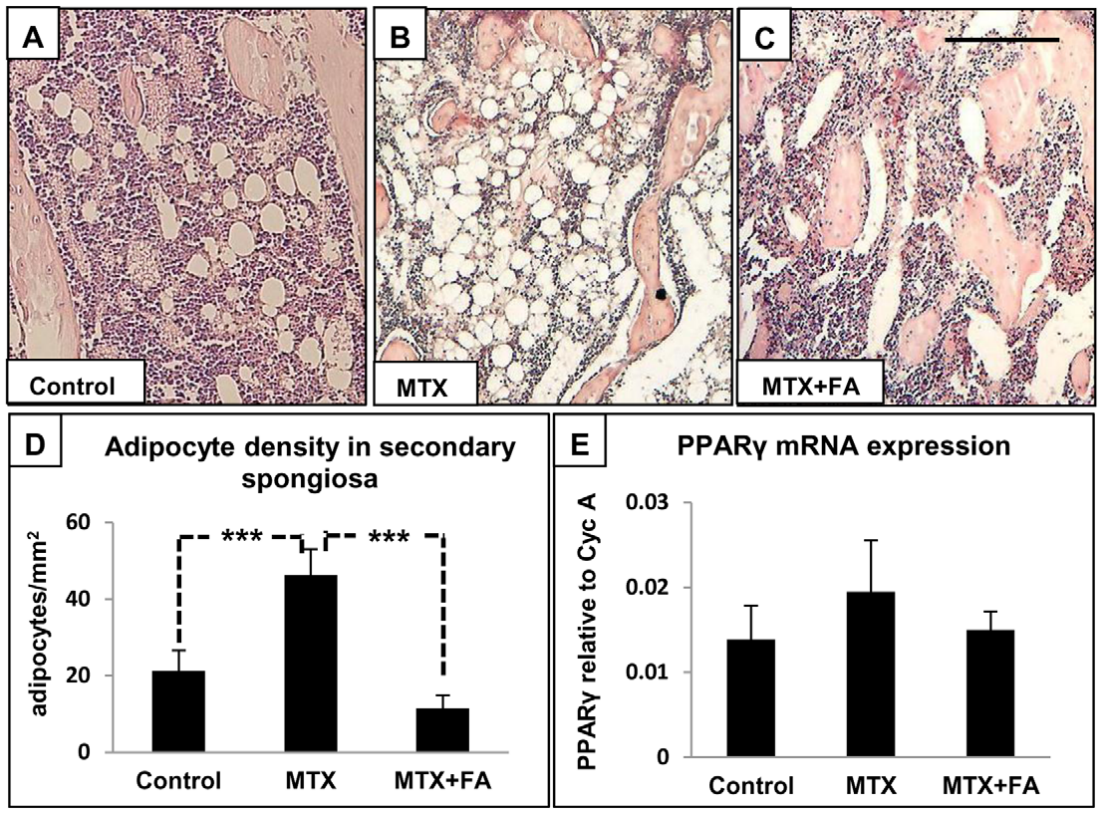
Effects of treatment with MTX alone or with supplementary folinic acid (FA) for 6 weeks on adipocyte density and mRNA expression of PPARγ. H&E-stained sections of tibia from a control (A), MTX-treated (B) and MTX+FA treated rat (C). Treatment effects on adipocyte density within bone marrow area of secondary spongiosa (D), and mRNA expression of PPARg relative to Cyclophilin-A (E). Scale bar on panel C = 250 µm, which applies to A and B.

To investigate whether long-term MTX treatment is associated with an increase in marrow adiposity, density of adipocytes was quantified within the bone marrow at the lower secondary spongiosa region on H&E stained tibia sections. This analysis revealed that MTX treatment caused a significant increase in adipocyte density within the bone marrow when compared to the normal control (P<0.001) **(** **Fig. 8A, 8B & 8D** **)**. However, FA supplementation was able to significantly suppress this adiposity phenotype (P<0.001) **(** **Fig. 8B, 8C & 8D** **)**. RT-PCR analysis revealed insignificant increased mRNA expression of adipogenic transcription factor PPARγ after MTX treatment which appeared to be suppressed by MTX+FA treatment **(** **Fig. 8E** **)**.

## Discussion

Due to the high success rate and intensifying use of chemotherapy in paediatric cancers, chemotherapy-induced bone growth defects, osteoporosis, osteonecrosis, and fractures are becoming major long-term adverse effects in young cancer patients and survivors. Despite many clinical and some animal studies [12], [14], [16] that have reported bone damage during and after chemotherapy, there is a lack of adjuvant treatments for protecting bone growth during childhood cancer chemotherapy. Using a chronic rat model of chemotherapy mimicking the clinical protocol with the commonly used anti-metabolite methotrexate (MTX), the current study observed that long-term high dose MTX chemotherapy reduces endochondral bone formation and increases bone resorption and marrow fat content, and that FA supplementation can prevent these adverse effects.

### Effects of Chronic Methotrexate Treatment on Growth Plate

Growth plate chondrocyte numbers and rates of their proliferation and apoptosis directly correlate to the longitudinal growth rate [22]. In the current study, long-term MTX chemotherapy caused a significant loss of growth plate chondrocytes and their columnar arrangement. To examine mechanisms of chondrocyte loss, the current study revealed that long-term high-dose MTX treatment caused no significant changes in chondrocyte proliferation. This is in contrast to previous studies that reported decreased growth plate cell proliferation in rats treated with high-dose MTX for a short-term (5 daily injections at 0.75 mg/kg) [13], [14]. However, consistent to the current study, a few studies have reported that MTX alone had no effects on proliferative response of chondrocytes both *in vivo* and *in vitro* [12], [23]. Similarly, while a previous study has demonstrated that 5 consecutive doses of MTX reduced collagen-II mRNA expression in growth plate of treated rats, the current study revealed that collagen-II expression in the growth plate was not significantly suppressed in rats receiving long-term MTX chemotherapy. These studies suggest that chondrocyte proliferation and collagen-2 expression appear to be more severely affected after acute consecutive daily MTX treatment in comparison to chronic twice weekly treatment, which may be due to differences in the cumulative drug effects with time.

Apart from appropriate chondrocyte proliferation, production of calcified cartilage scaffold for trabecular bone deposition also relies on appropriate balance of survival vs. apoptosis of chondrocytes. The current study revealed that chronic high-dose MTX treatment has a profound effect in inducing chondrocyte apoptosis at the lower proliferative and upper hypertrophic zones, where apoptosis is normally not observed, which is consistent with previous animal studies [14], [16]. Two major pathways are known to be involved in chemotherapy-induced apoptosis, including the death receptor pathway and mitochondrial dysfunction pathway [24]. The current study revealed consistent findings to one previous acute study, that long-term high-dose MTX treatment caused a notable induction of Fas and Fas-L expression [14]. Interestingly, high-dose corticosteroid treatment in rats was reported to promote apoptosis of chondrocytes and osteocytes via suppression of Bcl-2 and induction of Bax expression [25], suggesting different chemotherapeutic agents may induce apoptosis by different apoptotic pathways.

### Effects of Chronic Methotrexate Treatment on Metaphyseal Bone and Fat Contents

Mirroring the growth plate damages by chronic MTX chemotherapy, the current study further revealed a reduction in the thickness of primary spongiosa and reduced metaphyseal bone volume after chronic MTX treatment. µ-CT analysis also revealed that following the MTX treatment, bony trabeculae were fewer in number and more separated in spacing, a finding which is consistent with that reported in a short-term rat study [14].

The numbers and activities of osteoblasts and osteoclasts on the trabecular bone surface determine and modify the number, size and shape of bone trabeculae, influencing bone mass. In rats, short-term MTX administration was able to reduce osteoblast density by suppressing osteoblast and preosteoblast proliferation *in vivo* [14]. The current study revealed that chronic high-dose MTX treatment was able to decrease osteoblast density, which was found associated with the induction of osteoblast apoptosis rather than reduction in proliferation. However, quantitative RT-PCR analysis revealed a trend of increase in gene expression of osteogenic transcription factor osterix and major osteoblast protein osteocalcin in metaphyseal bone, suggesting a trend of increase in osteoblast maturation and probably representing a repair response for osteogenic potential following chronic high-dose MTX-treatment. Consistently, in a recent study, while there was a reduction in osteoblast density in rats treated chronically with low-dose MTX, the size of osteoprogenitor pool within bone marrow of the treated rats was increased [8]. This again suggested a trend of greater osteoblast differentiation potential within the bone marrow after long-term MTX chemotherapy. Interestingly, the increase in osteogenic potential during maintenance MTX chemotherapy (of a lower intensity) is in contrast with the decreased osteogenic potential of bone marrow stromal progenitor cells after 5 consecutive high-dose MTX treatment (representing the intensive induction phase) [26]. It is possible that the increased osteogenic potential after the long-term maintenance MTX treatment represents a recovery mechanism compensating for the damaged bone environment caused during the intense induction treatment phase.

Previous clinical studies have reported a low bone mass and an excess bone marrow content (adiposity) following the treatment of childhood ALL [27], [28]. Similarly, the phenomenon for reduced bone volume with increased marrow adiposity is also well described in ageing-related osteoporosis [29]. In the current study, accompanying the bone volume being reduced following long-term high-dose MTX administration, adipocyte density was found significantly increased within the metaphysis of treated rats, despite the increase in expression of adipogenic transcription factor PPARγ [30] not being statistically significant in the metaphysis. Previously, PPARγ has been identified to be a key transcriptional factor for adipocyte differentiation that regulates the lineage commitment of both marrow MSCs towards adipocytes and away from osteoblasts [31]. A recent study has demonstrated that acute intensive MTX treatment (equivalent to the induction phase of the current study) caused a significant switch of bone marrow stromal progenitor cells from osteogenesis to adipogenesis [26], with osteogenic transcription factors Runx2 and Osterix being decreased but adipogenic genes PPARγ and FABP4 being up-regulated in the stromal cell population. In the current study, the increased adipocyte density and reduced osteoblast density with unchanged expression of adipogenic and osteogenic regulatory genes could be related to the possibility of increased adipogenesis at the expense of osteoblastogenesis within the bone marrow occurring during the early intensive induction phase of MTX treatment as observed [26], but not during the maintenance phase. Mechanisms underlying MTX-induced potential bone/fat switch remain to be investigated.

### Effects on Metaphyseal Bone Resorption and Circulating Cytokines

Since the imbalance of bone formation and bone resorption processes causes skeletal morbidity, the current study also examined the treatment effects on bone resorptive osteoclasts and revealed that long-term MTX chemotherapy significantly increased osteoclast density on trabecular bone surface (2 folds of control). A recent *ex vivo* study using bone marrow cells obtained from rats treated with MTX for 5 days revealed an increase in osteoclast precursor (OCP) cell pool expressing preosteoclast surface marker CD11b^+^ [8], which is consistent with the observed increased osteoclast density on bone surface of treated rats [8]. Clinically, long-term glucocorticoid treatment is known to increase production of the major osteoclastogenic factor RANKL by osteoblasts, resulting in more osteoclast formation and bone resorption with a net loss of bone over time [32]. In the current study, however, gene expression analysis revealed a lack of significant changes in expression of RANKL/OPG ratio and in levels of osteoclastogenic cytokines (IL-6, TNF-α and IL-1β) in the metaphysis, indicating that expression of osteoclastogenic signals was not affected locally in bone during maintenance phase of MTX chemotherapy. This observation perhaps indicates that bone loss was mainly resulting from the more severe cellular and molecular damages during the intensive induction chemotherapy phase [14], [33], [34], in which the structural damages resulting from the initial intensive induction treatment may have persisted into the maintenance phase, where molecular recovery starts to take place in order to compensate for the damaged bone environment.

While levels of expression of osteoclastogenic signals were not affected locally in the metaphysis, blood plasma obtained from MTX-treated rats was shown able to induce more osteoclast formation *in vitro* in bone marrow cells from normal rats. Despite the results were not statistically significant, plasma from MTX-treated animals was able to induce more osteoclast formation than the positive control supported by exogenous osteoclastogenic growth factors (MCSF+RANKL), suggesting the systemic contribution of MTX chemotherapy to bone resorption. However, the extent of plasma contribution to osteoclast formation was found not as substantial as that observed in the acute model [19], suggesting that while the plasma systemic contribution to bone loss still remains during maintenance chemotherapy, the contribution and damages are greater during the intensive induction chemotherapy phase. In addition, the variation between plasma of MTX-treated animals in inducing osteoclast formation may also contribute to the lack of significant effects seen, a limitation that could be avoided by increasing the number of rat plasma per group in future studies.

Interestingly, protein levels of IL-1β were shown significantly elevated when compared to normal rat plasma. Furthermore, despite the statistical insignificance, the blunting effect of IL-1β neutralizing antibody in the plasma-induced osteoclast formation was observed in a dose-dependent manner, which suggests the importance of IL-1β in promoting osteoclast formation. However, the significant increase in IL-1β concentration within the plasma from MTX-treated rats did not closely reflect the extent of plasma-induced osteoclast formation, suggesting that the systemic IL-1β may only partially contribute to plasma-induced osteoclast formation. It is known that IL-1β promotes bone resorption and mediates bone resorption in inflammatory bone loss disorders. It participates in various stages of osteoclast development both *in vivo* and *in vitro*, including fusion of mononuclear pre-osteoclasts, potentiating osteoclast function and prolonging survival of osteoclasts [35], [36], [37]. IL-1β is secreted by osteoclast precursor (OCP) cells, but unlike TNF-α and RANKL, IL-1β cannot induce osteoclast formation directly from OCPs *in vitro* [38]. In addition to the known effect that IL-1β can induce osteoclast formation indirectly through up-regulation of RANKL via osteoblast/stromal cells [39], one recent study demonstrated that IL-1 can stimulate osteoclast formation directly from OCPs when they overexpress c-Fos, without addition of other cytokines [40]. Incidentally, MTX has previously been shown to increase the number of CD11b^+^ OCPs, which may provide an explanation of increased IL-1β as it is secreted by OCPs. In the study, without the up-regulation of RANKL protein within the systemic circulation after MTX treatment, plasma IL-1β appears to play a role in inducing more osteoclast formation. It could be possible that the MTX-induced increased OCPs may express a high level of c-Fos, therefore an increased level of systemic IL-1β could potentially induce osteoclast formation directly to mediate bone loss. However, further investigations are required to confirm this. Moreover, the unchanged RANKL protein levels in the plasma of MTX-treated rats do not mean RANKL is not required for osteoclast formation. RANKL is critical for osteoclastogenesis, and this study revealed that in the presence of RANKL (even without the upregulation of its expression), IL-1β upregulation within the circulation was able to induce more osteoclast formation. However, the possibility of other systemic factors contributing to bone loss cannot be ruled out.

### Prevention of Methotrexate-induced Bone Damage by Folinic Acid Supplementation and Clinical Relevance

Due to the high success rates in cancer treatment, MTX remains to be an important component of paediatric chemotherapy. MTX chemotherapy is associated clinically with bone pain, bone loss and increased fracture risks [41], and chronic use of MTX alone or in combination with other chemotherapy agents may lead to failure of BMD recovery even after discontinuation of treatment [42]. Therefore, it is necessary to develop potential strategies to protect bone growth during chemotherapy. Since folate is essential for cell proliferation and survival [43], [44], folate deficiency caused by repeated use of MTX could be one of the possible causes of skeletal defects. Folinic acid (FA) is an antidote that has been clinically used for supplementing MTX therapy to reduce hepatotoxicity and gastrointestinal side effects without lowering the efficacy of MTX [45]. Recently, FA treatment was found to protect against MTX-induced bone damage in short-term animal study [13]. The current study further revealed that FA supplementary treatment can diminish the damaging effects of chronic high-dose MTX at growth plate by preventing MTX-induced chondrocyte apoptosis via the suppression of pro-apoptotic molecules involved in the death receptor pathway. Thus FA supplementation preserved chondrocyte number and their columnar arrangement in MTX-treated rats. At the metaphysis, FA supplementation was able to preserve primary spongiosa heights and overall trabecular bone volume in MTX-treated rats; and this bone-protective effect was probably related to FA function in preventing osteoblast apoptosis (thus preserving osteoblast density) and preventing a higher density of osteoclasts (reducing bone resorption). Consistently, FA supplementation was previously shown to preserve osteoblast numbers and bone marrow stromal cell pool in rats receiving acute intensive MTX chemotherapy [13] and to suppress MTX-induced increased osteoclast density in rats treated with MTX at a high dose for a short term or at a low dose for a long-term [8]. While the current study revealed no significant changes in expression of genes involved in osteoclastogenesis in metaphyseal bone, FA supplementation was found to suppress the ability of plasma obtained from MTX-treated rats in supporting *in vitro* osteoclast formation. Interestingly, FA supplementation in blocking the ability of plasma from MTX-treated rats in inducing osteoclast formation was found associated with only partial abrogation of induction of IL-1β levels in the circulation, and the greater extent of reduction of osteoclast formation was not reflected by the insignificant lower level of IL-1β from the plasma of FA-supplemented rats. It is possible that while IL-1β may have contributed to osteoclastogenesis after MTX chemotherapy, effects of MTX+FA supplementary treatment on suppressing IL-1β and thus its likely role to the FA treatment-induced overall reduced osteoclast formation have been minimal. It is likely that FA may suppress MTX-induced osteoclastogenesis through other factors/cytokines (in addition to IL-1β) which will need to be addressed in future studies. In addition, FA may also act through other systemic mechanisms to suppress osteoclast formation. Clinical studies showed that FA administration thrice weekly can suppress serum malondialdehyde (S-MDA, a marker of oxidative stress and lipid peroxidation) [46], and oxidative stress is known to be associated with osteoporosis and other bone pathology. However, the mechanisms for systemic rescuing effect of FA in MTX chemotherapy require further investigation. Moreover, the current study revealed that FA supplementation can significantly reduce bone marrow adiposity resulted from MTX chemotherapy, indicating it may potentially preserve bone volume by preserving osteogenesis and suppressing osteoclastogenesis and adipogenesis. Present study lays the ground work for future investigation on whether FA prevents skeletal complications in childhood survivors and further scope to optimize the potential dual action of FA to reduce of chemotherapy induced soft tissue toxicity and skeletal complications.

### Conclusions

Using a chronic model in young rats mimicking the commonly used clinical protocol, the current study has identified that chronic high dose MTX chemotherapy causes bone growth defects by reducing endochondral bone formation and increasing bone resorption and marrow adiposity, and that folinic acid supplementation can prevent these adverse effects. While folinic acid treatment has been used in childhood chemotherapy to reduce toxicities of high dose MTX in soft tissues, our study suggests that folinic acid may be potentially useful in paediatric patients who are at risk of skeletal growth suppression and bone loss as a result of chronic high-dose MTX chemotherapy.
